# Interactions between free-living amoebae and *Cryptosporidium parvum*: an experimental study[Fn FN1]

**DOI:** 10.1051/parasite/2023033

**Published:** 2023-08-21

**Authors:** Marion Lefebvre, Romy Razakandrainibe, Damien Schapman, Arnaud François, Damien Genty, Ludovic Galas, Isabelle Villena, Loic Favennec, Damien Costa

**Affiliations:** 1 Univ Rouen Normandie, Laboratory of Parasitology-Mycology, EA7510 ESCAPE, University hospital of Rouen Normandie 76000 Rouen France; 2 National Reference Center Cryptosporidiosis, microsporidia and other protozoa, University Hospital of Rouen Normandie 76000 Rouen France; 3 Univ Rouen Normandie, INSERM, CNRS, HeRacLeS US 51 UAR 2026, PRIMACEN 76000 Rouen France; 4 Department of anathomopathology, University Hospital of Rouen Normandie 76000 Rouen France; 5 Reims Champagne-Ardenne University, Laboratory of Parasitology-Mycology, EA7510 ESCAPE 51454 Reims France

**Keywords:** *Cryptosporidium*, Free living amebae, Interactions, Phagocytosis, Infectivity, Biofilm

## Abstract

Free-Living Amebae (FLA) and *Cryptosporidium* oocysts occasionally share the same environment. From 2004 to 2016, *Cryptosporidium* was responsible for 60% of 905 worldwide waterborne outbreaks caused by protozoan parasites. The aim of this study was to evaluate interactions between *C. parvum* oocysts and two common FLAs (*Acanthamoeba castellanii* and *Vermamoeba vermiformis)* in a water environment. Encystment and survival of FLAs were evaluated by microscopy using trypan blue vital coloration. Oocysts were numerated on microscopy. Interactions were studied over time in conditions both unfavorable and favorable to phagocytosis. Potential phagocytosis was directly evaluated by several microscopic approaches and indirectly by numeration of microorganisms and oocyst infectivity evaluation. Occasional phagocytosis of *C. parvum* by FLAs was documented. However, oocyst concentrations did not decrease significantly, suggesting resistance of oocysts to phagocytosis. A temporary decrease of oocyst infectivity was observed in the presence of *A. castellanii*. The effect of these interactions on *C. parvum* infectivity is particularly interesting. The biofilm condition could favor the persistence or even the proliferation of oocysts over time. This study demonstrated interactions between *C. parvum* and FLAs. Further knowledge of the mechanisms involved in the decrease of oocyst infectivity in the presence of *A. castellanii* could facilitate the development of new therapeutic approaches.

## Abstract (fr)

Les amibes libres et les oocystes de *Cryptosporidium* partagent parfois le même environnement. Entre 2004 et 2016, *Cryptosporidium* a été responsable de 60 % des 905 épidémies d’origine hydrique dans le monde causées par des parasites protozoaires. Le but de cette étude était d’évaluer les interactions entre les oocystes de *C. parvum* et deux espèces d’amibes libres communes (*Acanthamoeba castellanii* et *Vermamoeba vermiformis*) en environnement aquatique. L’enkystement et la survie des amibes libres ont été évalués par microscopie en utilisant une coloration vitale au bleu trypan. Les oocystes ont été comptés au microscope. Les interactions ont été étudiées au cours du temps dans des conditions à la fois défavorables et favorables à la phagocytose. La phagocytose potentielle a été évaluée directement par plusieurs approches microscopiques et indirectement par la numération des micro-organismes et l’évaluation de l’infectiosité des oocystes. Une phagocytose occasionnelle de *C. parvum* par amibes libre a été documentée. Cependant, les concentrations d’oocystes n’ont pas diminué de manière significative, ce qui suggère une résistance des oocystes à la phagocytose. Une diminution temporaire de l’infectivité des oocystes a été observée en présence d’*A. castellanii*. L’effet de ces interactions sur l’infectiosité de *C. parvum* est particulièrement intéressant. La condition biofilm pourrait favoriser la persistance ou même la prolifération des oocystes de *C. parvum* au fil du temps. Cette étude a démontré des interactions entre *C. parvum* et amibes libres. Une meilleure connaissance des mécanismes impliqués dans la diminution de l’infectiosité des oocystes en présence d’*A. castellanii* pourrait faciliter le développement de nouvelles approches thérapeutiques.

## Introduction

*Cryptosporidium* is an intracellular protozoan parasite responsible for cryptosporidiosis disease in animals and humans. The associated global disease burden is high and there is currently no effective therapy. Cryptosporidiosis causes gastroenteritis characterized primarily by watery diarrhea, nausea, vomiting and abdominal pain [3]. In immunocompetent individuals, cryptosporidiosis is often self-limited but can evolve chronically, sometimes leading to post-infection irritable bowel syndrome or sometimes it is implicated in colon cancer [6, 24, 49, 50]. Regarding the most vulnerable patients, such as children and the immunocompromized, symptoms are more severe and could lead to death mainly due to dehydration [4]. According to the Global Enteric Multicenter Study (GEMS), *Cryptosporidium* is the second leading cause (5–15%) of moderate-to-severe diarrhea in infants in countries of sub-Saharan Africa and South Asia [34, 48]. Currently, a total of 42 species of *Cryptosporidium* have been described, infecting a wide variety of hosts. Among them, 20 species can infect humans. However, only two species clearly dominate human epidemiology: *C. parvum* and *C. hominis* [65]. These two species are responsible for more than 90% of human cases of cryptosporidiosis [9]. Species distribution is dependent on geography and socioeconomic conditions. *Cryptosporidium parvum* and *C. hominis* are equally distributed in industrialized nations such as in European countries, the United States, and Australia [27]. However, in France, *C. parvum* has a higher prevalence (72%) than *C. hominis* (24%). Conversely, *C. hominis* has been reported as the dominant species in developing countries, mainly due to direct contamination and poor hygiene conditions [46]. Contamination occurs by oocyst ingestion and transmission may be direct (person-to-person and animal-to-person) or indirect (ingestion of contaminated water or food) [16, 23]. The main difference is observed with direct transmission where *C. hominis* is mainly transmitted by interhuman contact (adults or children), whereas *C. parvum* is mainly transmitted by animal contact [3]. For immunocompetent individuals, the infectious dose 50% (ID50) was estimated at 132 oocysts for *C. parvum* [48, 65].

Regarding indirect transmission, water is often described as a vehicle for *Cryptosporidium* oocysts. For example, in a meta-analysis, the global prevalence of *Cryptosporidium* oocysts in investigated water was 36% with 25.7% in treated water, 40.1% in untreated water, 25.5% in drinking water and 7.5% in swimming pool water [9]. Oocysts are very resistant in the environment. Oocyst survival has been described for up to 18 months at +4 °C and up to 7 months at +15 °C in water [25]. To inactivate *Cryptosporidium* oocysts, the temperature must exceed +72.4 °C for at least one minute or +64.2 °C for 5 min, or drop below −70 °C for at least one hour [13]. In addition, oocysts are resistant to water disinfection treatment, especially chlorine [33]. It has been shown that to inactivate 99% of oocysts they had to be exposed to 80 mg/L of free chlorine for 2 h, and to inactivate 100% of them, they needed to be exposed to 8 to 16 g/L of free chlorine for 24 h [33, 53]. This could at least partially explain why *Cryptosporidium* has frequently been reported as responsible for waterborne outbreaks. Between 2011 and 2016, *Cryptosporidium* was responsible for 63% of reported outbreaks due to protozoan waterborne transmission and was reported as the second leading cause of diarrheal disease and death in children in developing countries [12, 15, 47]. The most cited outbreak of cryptosporidiosis occurred in Milwaukee in 1993. More than 400,000 people were infected after contamination of the drinking water system, leading to 4,000 hospitalizations and 69 deaths [39]. Between 2009 and 2017, in the United States, 444 cryptosporidiosis outbreaks were reported and 183 (41.2%) were of water origin. Contaminated recreational water was also involved in cryptosporidiosis outbreaks in England and Wales (46%) [5]. In summary, water is undeniably a favorable environment for oocyst dissemination and survival, as for many microorganisms. This could lead to interesting interactions between microorganisms. Among them, free-living amebae (FLA) must be considered. FLA are protozoan parasites, ubiquitous in hydric and telluric environment [1, 35]. They have been found in 20–30% of domestic tap water samples [52] and in 68.9% of hospital samples [37]. Two FLA species are predominant in the environment: *Acanthamoeba castellanii (A. castellanii)* and *Vermamoeba vermiformis (V. vermiformis)*. *Acanthamoeba castellanii* has been extensively studied in the literature; however, it has been shown that the density of *V. vermiformis* in water and in biofilm was higher than that of *A. castellanii* and in particular in hot water systems [11, 43, 45, 57, 60]. Their prevalence in water networks is associated with biofilms. Biofilms serve as feeding grounds for FLA, which play a role in the reduction of bacterial biomass thanks to phagocytosis [1, 58].

Free-living amebae present two developmental stages, a vegetative feeding stage (trophozoite), and a resistant stage (cyst) that provides protection from harsh environmental conditions, such as changes in temperature, pH or even biocides and disinfectant exposure [17]. Encysted FLA can survive at least 24 years at +4 °C in water [41, 58] or over 20 years in a completely dry environment [54, 58]. In addition, FLA are very resistant to halogenated treatments (chlorine, bromine, iodine) widely used for water and surface disinfection [29, 55]. For example, a concentration of 15 mg/L of free chlorine was necessary to eliminate more than 4 log10 cysts of *V. vermiformis* and 2,500 mg/L to eliminate between 2 and 6 log10 cysts of *Acanthamoeba* sp. [7, 14]. Only four species of FLA have been described as direct human pathogens: *Acanthamoeba* spp., *Naegleria fowleri*, *Balamuthia mandrillaris* and *Sappinia diploidea* [35, 59]. FLA are responsible for severe infections mainly due to treatment resistance. For example, FLA keratitis frequently leads to blindness [38] and granulomatous amebic encephalitis to hemorrhagic necrosis of the central nervous system [1]. Another example is *Naegleria fowleri,* which is responsible for primary amebic meningoencephalitis in immunocompetent individuals [1], resulting in a rare acute fulminant infection of the central nervous system with fatal outcome. These organisms are also indirectly implicated in human disease due to their ability to serve as hosts for other pathogens [10]. Indeed, FLA are able to phagocytize a large variety of microorganisms, but some microorganisms, known as Amoeba-Resisting Bacteria (ARB) are able to resist phagocytosis. By serving as host for ARB, FLA play a protective role and facilitate the dissemination of ARB in the environment. Bacteria such as *Legionella* sp. or *Listeria monocytogenes* multiply inside the amoeba before being disseminated in the environment [19]. Out of 539 reported bacterial pathogenic species in humans and/or animals, 18.9% (102) were described as ARB [58]. Interactions with FLA were also reported with viruses (Adenoviridae, Pithovirus, etc.), fungi (*Cryptococcus neoformans, Candida* sp., etc.) or protozoa (*Toxoplasma gondii*) [1, 22, 62].

The aim of this study was to investigate the potential interactions between *C. parvum* oocysts and two common species of FLA: *A. castellanii* and *V. vermiformis* in water. Studied conditions were selected to be both unfavorable and favorable for phagocytosis to facilitate observation of potential interaction due to phagocytosis. Hypotheses were: (i) FLA phagocytise *Cryptosporidium* oocysts and consequently could be used as predators to manage *Cryptosporidium* contamination in the environment, or (ii) oocysts resist FLA phagocytosis. To the best of our knowledge, such interactions have never been investigated in water in such conditions, using both microscopy and infectivity evaluation.

## Materials and Methods

### Strains

*Cryptosporidium parvum* strains were obtained from successive batches of feces from infected calves (from the National Institute of Agricultural Research, Nouzilly, France). More precisely, oocysts were isolated by ImmunoMagnetic Separation (IMS) using the Isolate for IMS of *Cryptosporidium* oocysts kit (TCS Biosciences, Buckingham, UK), according to the manufacturer’s recommendations. Purified oocysts were stored at +4 °C in sterile phosphate-buffered saline (PBS) solution (Gibco^TM^, Thermo Fisher Scientific, Courtaboeuf, France) until use.

*Acanthamoeba castellanii* (ATCC 30234) and *V. vermiformis* (ATCC 50803) strains were cultured axenically in 25 cm^2^ flasks (Falcon^®^, Dutscher, Issy-les-Moulineaux, France) in sterile Peptone-Yeast extract-Glucose medium (PYG) and incubated at +28 °C. The PYG medium contained for 1 L of distilled water: 20 g proteose-peptone (BD Difco, Temse, Belgium), 1 g yeast extract (BD Difco), 0.98 g MgSO_4_,7H_2_O, 1 g sodium citrate, 2H_2_O, 0.02 g Fe(NH_4_)_2_(SO_4_)_2_, 6H_2_O, 0.34 g KH_2_PO_4_, 0.394 g NA_2_HPO_4_, 7H_2_O, 9 g glucose and 0.059 g CaCl_2_ according to the protocol described by Coulon *et al.* [8]. After 5 days of incubation at +28 °C, suspensions of FLA were obtained by scraping the flasks using a cell scraper (Nunc^TM^ Thermo Fisher Scientific). After centrifugation (1000G for 5 min), the pellet was washed once and suspended in 3 mL of sterile water before counting on a kova-slide (Dutsher).

The ATCC 27853 strain of *Pseudomonas aeruginosa* and a clinical strain of *Escherichia coli* were used. The bacterial strains were cultured on blood agar (Bio-Rad, Marnes-la-Coquette, France) and incubated at room temperature before use. Every 7 days, the *P. aeruginosa* and *E. coli* strains were subcultured.

### Unfavorable phagocytosis condition interactions

*Acanthamoeba castellanii* or *V. vermiformis* and *C. parvum* were co-incubated in sterile water for several days. We used sterile water to avoid any other potential interaction of microorganisms. Suspensions were incubated at +8 °C (±4 °C) for 28 days in a falcon tube (Falcon^®^, Dutscher). Each of the strains were incubated at the concentration of 10^6^ cells/mL (v/v) with a Multiplicity Of Infection (MOI) = 1. Interactions were evaluated overtime (0 (3 h), 3, 7, 14, 21 and 28 days). Observations were done at each sampling point after resuspension of solutions by vortex agitation (for one minute). Observations were done: (i) microscopically for numeration and viability assays of FLA (using trypan blue 0.4% (v/v) (Gibco^TM^, Thermo Fisher Scientific); only viable cells were considered for figure representations (trophozoites + cysts)); ii) microscopically for numeration of *C. parvum* oocysts, and iii) by cell culture coupled with qPCR (CC-qPCR) to evaluate infectivity of *C. parvum* oocysts, as described below. Three replicates per condition were done with at least six observations per replicate.

### Favorable phagocytosis condition interactions (biofilm condition)

One mL of a suspension of optical density at 0.5 McFarland of *P. aeruginosa* was inoculated in 24-well plates (Thermo Fischer Scientific, Roskilde, Denmark). After 24 h of incubation at room temperature, supernatant was removed and each well was washed three times using sterile water. Presence of biofilm was confirmed in reverse microscopy for each well constituting a support to both *Cryptosporidium* and FLA and theoretically to promote phagocytosis. Suspensions of FLA and/or *C. parvum* oocysts were inoculated in biofilm coated wells at a quantity of 5 × 10^5^ cells and incubated at room temperature for a maximum of 7 days. For each sampling point (3 h, day 1, day 3 and day 7), wells were rinsed three times with sterile water; then, biofilms were scraped in 500 μL of sterile water and transferred to 1.5 mL Eppendorf tubes (Eppendorf™, Thermo Fisher Scientific). Microbial interactions were evaluated as described above. The biofilm control condition corresponded to observations done immediately (<15 min) after microorganism inoculation. Three replicates per condition were done with at least six observations per replicate.

### Imagery used to observe potential phagocytosis

#### Confocal microscopy

On MatTek’s 35 mm glass bottom plates (MatTek corporation, Ashland, MA, USA), a biofilm of *Pseudomonas aeruginosa* DO: 0.5 was formed over 24 h. Then, the biofilm was inoculated with *A. castellanii/V. vermiformis* and *C. parvum* at the same concentration (5 × 10^5^ cells) for 1 and 3 h (most favorable times of interactions, see Results section) at room temperature. For each sampling point, the microorganisms were labelled according to the following protocol: (i) air drying of plates, (ii) addition of methanol, (iii) air drying, (iv) PBS washing, (v) DAPI (4′,6-diamidino-2-phenylindole) (Thermo Fisher Scientific) labelling at 1:500 dilution to mark amebae as well as sporozoites contained in *C. parvum* oocysts (10 min at +37 °C), (vi) PBS washing, (vii) labelling with 1/3 diluted Crypto-Cell-FITC (TCS Biosciences) to mark the outer wall of oocysts (15-minute incubation at +37 °C in humid atmosphere), (viii) PBS washing, (ix) storing at +4 °C in 2 mL of PBS. Samples were then observed using a Leica TCS SP8 confocal laser-scanning microscope (Leica Microsystems, Wetzlar, Germany). Images were acquired with an oil immersion objective (×63) with a numerical aperture of 1.4. Sequential acquisition was performed to avoid the overlapping emission spectra of the fluorescent markers and to increase the quality of these images. DAPI and Crypto-Cel-FITC were excited at 405 and 488 nm, respectively and their fluorescence emissions were collected between 425 and 475 nm for DAPI and between 490 and 530 nm for Crypto-cel-FITC on a photon counting detector (HyD, Leica Microsystems). A z-stack acquisition was performed to obtain a 3D volume of samples. We used a 250 nm z-step-size to avoid photobleaching and to optimize the acquisition of samples. Image processing (Maximum Intensity Projection, Merging channels, Brightness and Contrast adjustment) was performed with ImageJ.

#### Video

The interactions between *C. parvum* and *A. castellanii* were observed on video. Microorganisms were incubated at a concentration of 10^5^ cells/mL (MOI = 1) in MatTek dishes and observed on the CellDiscoverer 7, Zeiss video microscope. Videos were obtained with a water immersion objective (x 50).

#### Transmission electronic microscopy (TEM)

A control condition was done via suspensions containing exclusively 2 × 10^6^ oocysts/mL or 10^6^ amebae/mL. Then, to evaluate the potential phagocytosis, observations were done in the favorable phagocytosis condition where 4 × 10^6^ cells/mL of *Cryptosporidium* oocysts and FLA (MOI = 1) were co-incubated for 3 h in the bacterial biofilm. The biofilm was previously formed in 24-well plates, as described above. After 3 h of incubation, samples were fixed with glutaraldehyde in 0.1 M phosphate buffer pH 7.4 (±0.2) and post-fixed in osmium tetroxide. Samples were then subjected to a dehydration step with ethanol baths, followed by an impregnation step with Epoxy resin and then an inclusion and polymerization step leading to the formation of resin blocks. These resins blocks were then cut into semi-fine (0.8–1 μm) and ultra-fine (65–90 μm) sections, which were collected on copper grids and contrasted by uranyl acetate and lead citrate. The copper grids were observed in TEM (CM 10 microscope, Philips).

### Cell culture (used for evaluation of oocyst infectivity)

Human ileocecal adenocarcinoma HCT-8 cell lines (ATCC CCL-224) were used for cell culture investigations. HCT-8 cells were maintained in Rosewell Park Memorial Institute « RPMI » 1640 medium with glutamine (Lonza, Verviers, Belgium) supplemented with 5% of heat-inactivated fetal bovine serum (Eurobio, Les Ulis, France), 100 IU/mL of penicillin (Corning™; Thermo Fisher Scientific) and 100 pg/mL of streptomycin (Corning™; Thermo Fisher Scientific). Cultures were grown in Falcon flasks (75 cm^2^) (Falcon^®^, Dutscher) at +37 °C and 5% CO_2_ atmosphere. HCT-8 cells were resuspended twice a week until use.

### Infectivity evaluation of *C. parvum* oocysts

To evaluate the infectivity of oocysts, we adapted a qPCR method combined with HCT-8 cell culture (CC-qPCR) previously published by Kubina *et al*. [36]. Briefly, HCT-8 cells were cultured in 96-well plates (Thermo Fischer Scientific, Roskilde, Denmark) (2 × 10^4^ cells per well) at +37 °C with 5% CO_2_ to obtain about 90% confluence after 72 h of culture. Confluent HCT-8 cells were then infected with a co-culture medium containing *C. parvum* oocysts (10^3^ oocysts per well) for 48 h. In parallel, wells without HCT-8 cells were inoculated with *C. parvum* oocysts in the same conditions. DNA was extracted using a QIAamp DNA mini kit (QIAGEN, Hilden, Germany), according to the manufacturer’s recommendations. DNA was then quantified by qPCR (primers: Crypto-F: 5′–CGCTTCTCTAGCCTTTTCATGA–3′, CRYPTO-R: 5′–CTTCACGTGTGTTTGCCAAT–3′ and probe CRYPTO P: FAM 5′–CCAATCACAGAATCATCAGAATCGACTGGTATC–3′ BQH1), according to the following PCR program: 95 °C for 3 min; 95 °C for 15 s, and 60 °C for 60 s, repeated 45 times. Differences of obtained cycle threshold (Ct) values (named DeltaCt) were calculated subtracting Ct values obtained from infected wells without HCT-8 cells from Ct values obtained from infected wells with HCT-8 cells. Due to viable oocyst replication on HCT-8 infected cells, higher DeltaCt values were associated with higher proliferation of oocysts. Three replicates per condition were done with at least six observations per replicate.

### Evaluation of potential chemical interactions on infectivity of oocysts

Results showed decreased infectivity of oocysts in 3 h of contact between *A. castellanii* and *C. parvum* (see below). To study the effect of exclusive chemical contact between *C. parvum* and *A. castellanii,* we used a 5 μm of porosity cell insert (Merck Millipore 051715B, Molsheim, France). Suspensions of sterile water loaded with 10^5^ *C. parvum* oocysts were inoculated in a 24-well plate. Additionally, inserts were loaded with *A. castellanii* sterile water suspensions at the same concentration. Loaded inserts were dropped in wells previously inoculated with oocysts to block physical contact between *C. parvum* oocysts and *A. castellanii* while allowing chemical interactions. After 3 h of incubation at room temperature, inserts were removed and suspensions of oocysts were recovered from wells. A CC-qPCR was performed to evaluate oocyst infectivity, as previously described.

### Statistical analysis

Student’s test was used to compare the data. For a *p*-value < 0.05, the data were considered significantly different.

## Results

### Numeration of *C. parvum* oocysts and free-living amebae

**Figure 1:**
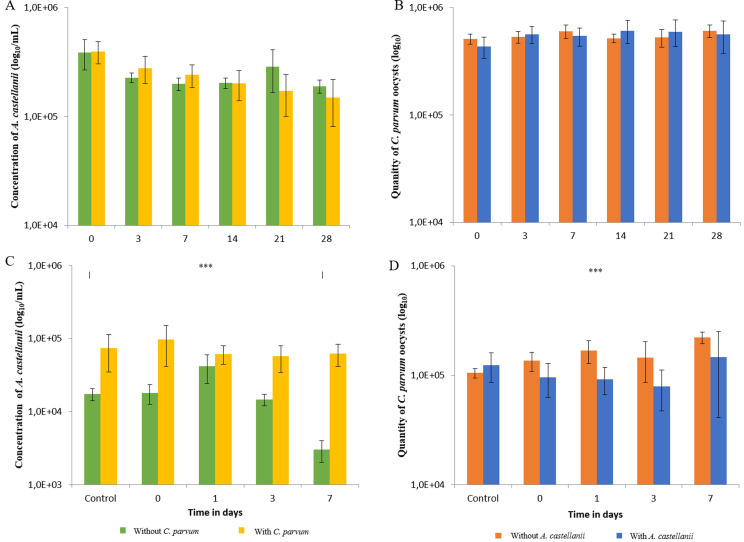
Enumeration of *Acanthamoeba castellanii* in the presence or absence of *Cryptosporidium parvum* oocysts over time in the unfavorable phagocytosis condition (1A) and in the favorable phagocytosis condition (1C) and enumeration of *C. parvum* oocysts in the presence or absence of *A. castellanii* over time in the unfavorable phagocytosis condition (1B) and in the favorable phagocytosis condition (1D). *p*-value: *** < 0.001. (magnification ×200 for *A. castellanii* and ×400 for *C. parvum*).

In the unfavorable phagocytosis condition, the concentration of *A. castellanii* decreased from 5.58 ± 0.11 log_10_/mL at D0 (Day 0) to 5.12 ± 0.18 log_10/_mL at D28 in the presence of *C. parvum* oocysts (*p*-value < 0.001). In the absence of *C. parvum* oocysts, *A. castellanii* decreased from 5.56 ± 0.13 log_10_/mL at D0 to 5.27 × 0.06 log_10_/mL at D28 (*p*-value < 0.001) (Fig. 1A). Regarding each sampling point, no significant differences were observed according to the presence or absence of *C. parvum* oocysts. Similar results were obtained with *V. vermiformis* (data not shown)*.* The concentration of *C. parvum* oocysts remained constant over time and was not significantly affected by the presence of *A. castellanii* or *V. vermiformis* (Fig. 1B).

In the favorable phagocytosis condition, the concentration of *A. castellanii* decreased over time from 4.22 ± 0.08 log_10_ to 3.41 ± 0.21 log_10_ in the absence of *C. parvum* oocysts (*p*-value < 0.001). In the presence of *C. parvum* oocysts, the concentration of *A. castellanii* remained stable over time in the studied conditions (Fig. 1C). Significant differences were observed according to the presence or absence of *C. parvum* oocysts at each studied sampling point. For *V. vermiformis* the results were similar (data not shown)*.* In the presence of *A. castellanii*, the concentration of *C. parvum* remained stable over time but, in the absence of *A. castellanii*, the concentration of oocysts increased significantly (but slightly) from 5.02 ± 0.04 log10 to 5.34 ± 0.06 log_10_ (a global two-fold increase) (*p*-value < 0.001) (Fig. 1D). Similar results were observed with *V. vermiformis.*

### Encystment of free-living amebae

**Figure 2:**
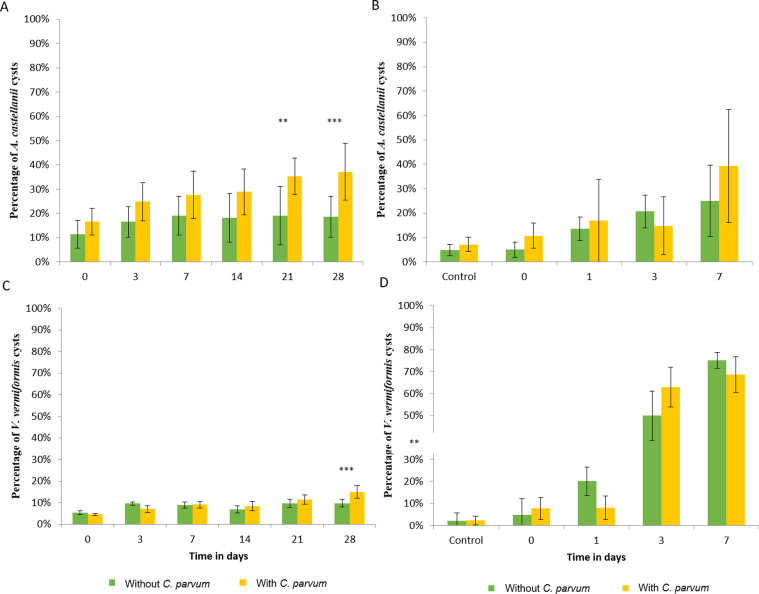
Percentage of *Acanthamoeba castellanii* cysts in the unfavorable phagocytosis condition (2A) and in the favorable phagocytosis condition (2B) and *Vermamoeba vermiformis* cysts in the unfavorable phagocytosis condition (2C) and in the favorable phagocytosis condition (2D) over time in the presence or absence of *Cryptosporidium parvum* oocysts. *p*-value: ** < 0.01; *** < 0.001.

Regarding encystment of *A. castellanii* in the presence of *C. parvum* oocysts (Fig. 2): in the unfavorable phagocytosis condition, the percentage of cysts tended to increase over time from 16.61% ± 5.47% to 37.16% ± 11.72% (*p*-value < 0.001). In the absence of *C. parvum* oocysts, encystment increased to a lesser extent, ranging from 11.43% ± 5.66% to 18.68% ± 8.41% (*p*-value < 0.05). Results were similar in the favorable phagocytosis condition (Fig. 2B). Similarly, the percentage of *V. vermiformis* cysts in the favorable phagocytosis condition, increased significantly over time (*p*-value < 0.001). Co-incubation with oocysts did not change encystment at the different sampling points (Fig. 2D). Regarding the unfavorable phagocytosis condition, in the presence of oocysts, the proportion of cysts increased from 4.53% ± 0.58% to 14.92% ± 2.92% (*p*-value < 0.001) and in the absence of oocysts from 5.4% ± 0.98% to 9.78% ± 1.86% at D28 (*p*-value < 0.001) (Fig. 2C).

### Infectivity of *C. parvum* oocysts

**Figure 3:**
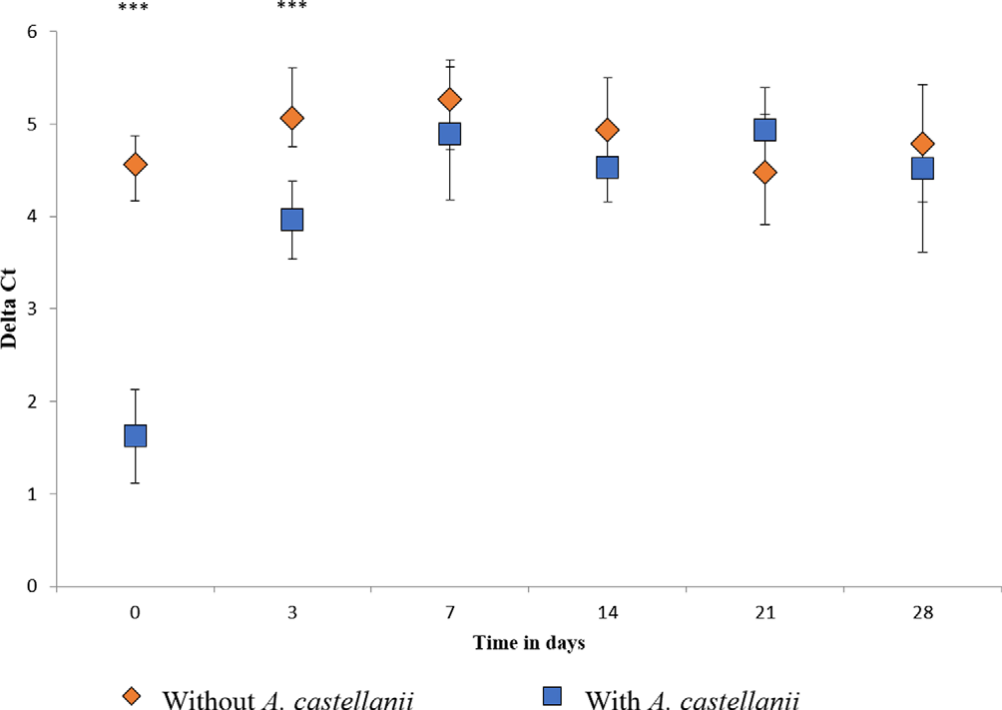
Evaluation of the infectivity of *Cryptosporidium parvum* oocysts over time in the presence of *Acanthamoeba castellanii*. *p*-value: *** < 0.01.

Results showed a significant decrease of Delta Ct in the early stages of co-incubation (Fig. 3) in comparison with the control condition (in the absence of *A. castellanii*). This suggests an early loss of infectivity in the presence of *A. castellanii*. Additional results showed a maximum decrease of oocyst infectivity at 3 h of co-incubation in the presence of *A. castellanii* (data not shown).

Results also showed that when *A. castellanii* and *C. parvum* oocysts were not in physical contact (separated by the insert), the infectivity of oocysts did not change in the presence of *A. castellanii*.

### Phagocytosis evaluation

**Figure 4:**
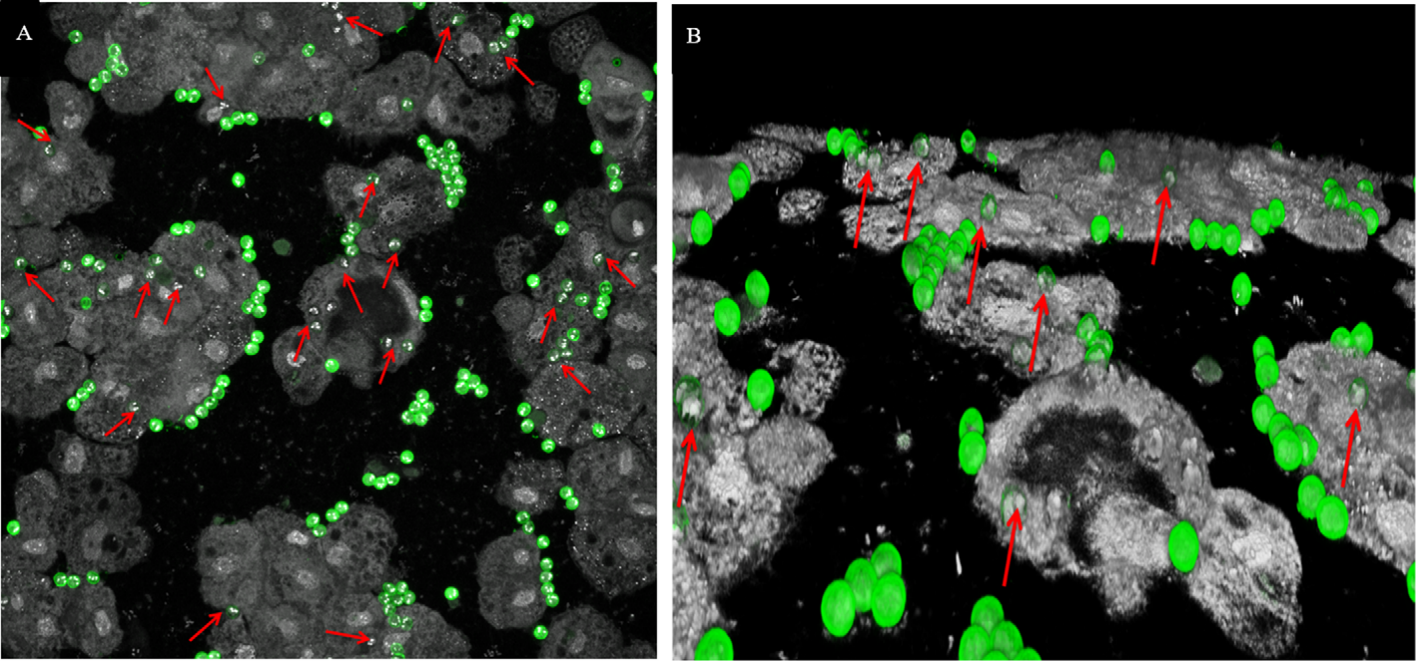
Confocal imaging (A) and 3D projection (B) of co-incubated free-living amebae and *Cryptosporidium parvum* oocysts (green). Red arrows point to oocysts internalized in FLA.

A video was made to evaluate interactions between *C. parvum* oocysts and *A. castellanii* (Supplemental file 1). In the video, we can see favorable tropism of *A. castellanii* in the direction of *C. parvum* oocysts. Oocysts seemed sometimes to be incorporated into FLA, but were always released over time. Confocal imaging and 3D projection showed *C. parvum* oocysts occasionally inside *A. castellanii* or *V. vermiformis* (Figs. 4A and 4B). Interestingly, confocal imaging also showed *C. parvum* oocysts frequently clustered around FLA.

**Figure 5:**
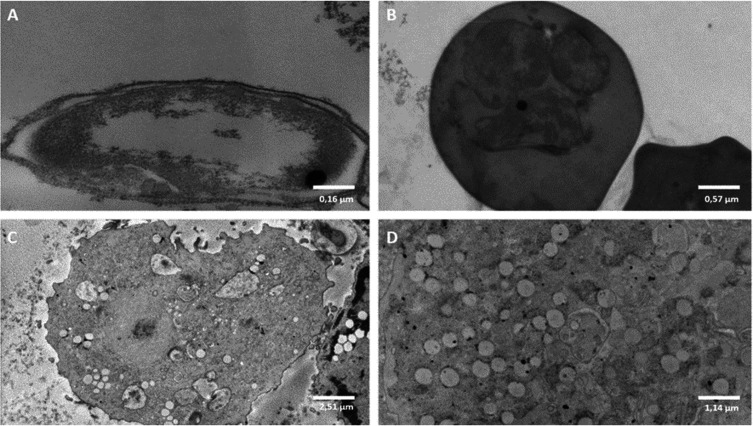
Images obtained from transmission electronic microscopy (TEM) of *Cryptosporidium parvum* sporozoites (5A), *C. parvum* oocysts (5B) and vegetative forms of *Acanthamoeba castellanii* (5C and 5D).

To complete the investigation, TEM was performed. First, each microorganism was observed from the monomicrobial condition (Fig. 5). Sporozoites and oocysts of *Cryptosporidium parvum* are shown in Figures 5A and 5B, respectively. Vegetative forms of *A. castellanii* are shown in Figures 5C and 5D.

**Figure 6:**
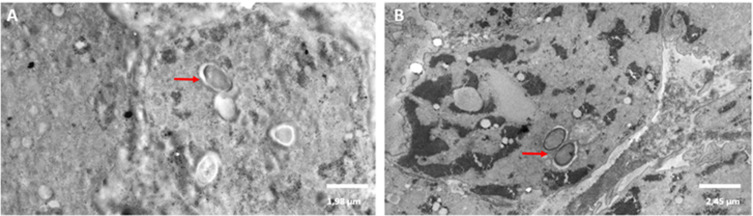
Transmission electron microscopy (TEM) observations of vegetative forms of *Acanthamoeba castellanii* co-incubated with *Cryptosporidium parvum*. Red arrows show sporozoites inside trophozoites of *A. castellanii* (6A and 6B).

In co-incubation condition, sporozoites were observed inside *A. castellanii* (Figs. 6A and 6B). In addition, “digestive-like vacuoles” (5–8 μm) were sometimes observed inside *A. castellanii* trophozoites (data not shown).

## Discussion

This study evaluated potential interactions between FLA and *Cryptosporidium* oocysts using both microscopy (optical, confocal, fluorescent, and TEM) and qPCR (for infectivity evaluation) approaches. Until now, very few data have been published on interactions between FLA and *Cryptosporidium* oocysts [18, 51, 56]. De Moraes *et al.* studied growth, encystment, and survival of *Acanthamoeba castellanii* in the presence of various bacterial species (*E. coli, P. aeruginosa, Enterobacter cloacae, Bacillus megaterium, Micrococcus luteus* and *Staphylococcus aureus*) in non-nutrient saline solution. They demonstrated a dose-dependent proliferative response of *A. castellanii* according to bacterial concentration. Proliferation of *A. castellanii* (6–8 fold increase) was observed after addition of 10^9^ bacteria/mL corresponding to an MOI (bacteria: FLA) of 10 000:1 [42]. In the absence of bacteria, *A. castellanii* cells stopped dividing but remained viable and retained their typical trophozoite morphology for 4 to 7 days and thereafter began to encyst [42]. In our study, FLA did not proliferate in the presence of oocysts, with an MOI of 1:1. A higher MOI, with a higher ratio of oocysts to FLA could potentially lead to FLA proliferation over time. However, a higher MOI could not be easily reached due to the impossibility to culture oocysts *in vitro*, in contrary to bacteria or yeast for example. One possibility to obtain more oocysts is to work in animal models (requiring many animals), with all corresponding ethical aspects. In addition, lowering the concentration of FLA to obtain a higher MOI would strongly limit observations by microscopy. However, it has also been shown that for an MOI of 1:1 (10^6^/mL bacteria and *A. castellanii*), bacteria (*P. aeruginosa*, *E. coli, Serratia marescens* and *Stenotrophomonas maltophilia*) favored the growth and survival of *A. castellanii* and that for a higher MOI of 100:1 (bacteria: FLA), bacteria and especially *P. aeruginosa* inhibited the growth of *A. castellanii* [40, 61]. Indeed, FLA proliferation occurs in the presence of various bacteria and FLA have preferences for certain bacterial species and in particular gram-negative bacteria such as *E. coli* or *Klebsiella aerogenes* [28]. In addition, it has been reported that FLA cultured in axenic conditions could not predate bacteria such as *Acinetobacter baumanii*. Axenic growing conditions could thus favor pinocytosis rather than phagocytosis and consequently minimize bacterial interactions [2, 31]. In our study, the FLA used were axenically cultured for years, which may affect their phagocytosis ability and potentially limit oocyst interactions.

Interestingly, it has been shown that temperature influences the phagocytosis ability of FLA. After ingestion at +20 °C, *Legionella pneumophila* was fully digested by *A. castellanii* but at +37 °C, *Legionella pneumophila* could lyse *A. castellanii* [40]. *Parachlamydia acanthamoebae* was lytic for *Acanthamoeba polyphaga* between +32 and +37 °C and endosymbiotic between +25 °C and +30 °C [19]. In our study, interactions were first studied in an unfavorable phagocytosis condition, in a suspension at +8 °C (±4°C). And then, we evaluated interactions in a most favorable phagocytosis condition at room temperature (+20 °C to +25 °C) in biofilm. Since *A. castellanii* rapidly decreased in the favorable phagocytosis condition in the absence of *C. parvum* oocysts and encystment increased over time in the presence of oocysts, results seem to demonstrate that oocysts could delay the decline of *A. castellanii*. This phenomenon was described with bacteria, which delayed the encystment of *A. castellanii* (7–8 days in absence of bacteria vs. 9–16 days) and improved the encystation yield (0.5 in absence of bacteria *versus* 4–44 depending on the bacterial strain) [42]. In addition, encystment is dependent on the FLA strains studied and probably on their resistance. Both temperature and desiccation could, at least partially, explain such differences. *Vermamoeba vermiformis* seemed more sensitive to desiccation than *A. castellanii*. In the literature, it has been shown that FLA encyst to fight against desiccation and that *Acanthamoeba* spp. cysts can survive for up to 20 years in a completely dry environment [54]. Interestingly, in our studied conditions, macroscopic desiccation was observed after 7 days of incubation, corresponding to the highest observed encystment rate.

In our studied biofilm condition, very surprisingly, the number of *C. parvum* oocysts increased progressively over time (factor 2) in the absence of FLA. In the presence of FLA, such proliferation of oocysts was not observed. Some hypotheses could explain the observed differences: in the absence of FLA, *C. parvum* oocysts could possibly interact with the bacteria supporting the biofilm (i.e., *P. aeruginosa*). In the literature, a 2–3 fold multiplication of oocysts was shown in a mature biofilm of *P. aeruginosa* in 6 days, together with an observation of different developmental stages of *Cryptosporidium* (sporozoites, trophozoites, and type I and II meronts) [32]. Conversely, other studies showed that the number of oocysts remained constant in biofilm conditions [63, 64]. These discrepancies are possibly related to the difference in biofilm type. The first study used an artificial biofilm of *P. aeruginosa*, and the second used a natural biofilm sampled from runoff water. As artificial and natural biofilms have different communities, structures, and nutrient levels, oocysts may interact differently. Furthermore, several studies have reported the possible multiplication of *Cryptosporidium* extracellularly in cell-free cultures showing that encapsulation in a host cell was not essential for oocyst multiplication [20, 21, 26, 66].

Finally, regarding infectivity of oocysts, a temporary decrease of infectivity was observed in the presence of *A. castellanii*. Investigations revealed that physical interactions between oocysts and *A. castellanii* seemed mandatory for such effect. Thus, the decrease of *Cryptosporidium* infectivity could be linked to FLA attempted phagocytosis. At the beginning of the co-incubation, *Cryptosporidium* could develop escape mechanisms against FLA predation leading to a reduction in its infectivity. Although no proof of oocyst phagocytosis was observed with TEM (even though we observed potential digestive vacuoles similar in size to oocyst vacuoles), TEM observations demonstrated sporozoite phagocytosis by FLA. Consequently, phagocytosis could preferentially occur in “free” forms of *C. parvum*, explaining why oocyst numeration does not decrease over time in the presence of FLA and why no increase of oocysts was observed in the biofilm condition in the presence of FLA. In addition, SP8 microscopy showed clusters of oocysts attached to FLA membranes (probably not observed by conventional light microscopy because the microorganisms in solution were vortexed before microscopic study). This could be due to the traction effect of FLA on oocysts; however, groups of oocysts were also observed away from FLA. In the literature, agglutination of *Cryptosporidium* oocysts has been observed in raw water, whereas in sterile water, no agglutination was observed. This phenomenon may be a response to predation [30]. This defense mechanism has already been reported for *P. aeruginosa*. Pickup *et al.* observed the formation of bacteria microcolonies, leading to a reduction in the rate of bacteria ingestion by FLA [44]. Other mechanisms of resistance to FLA predation have been described in the literature, such as the presence of a polysaccharide capsule (i.e., *C. neoformans*). *Cryptosporidium parvum* oocyst walls could similarly limit FLA phagocytosis.

This study provides new data on interactions between FLA and *C. parvum* oocysts, including both microscopy and infectivity data. FLA survival was increased in the presence of *C. parvum*. Various microscopic observations demonstrated the ability of FLA to occasionally phagocytize *C. parvum*. Interestingly, *C. parvum* was able to resist phagocytosis, but its infectivity was temporarily modified. Physical interactions between *A. castellanii* and oocysts appeared essential in the corresponding mechanism of modified infectivity. These results open new perspectives based on *Cryptosporidium* microbial interactions and the possibility to modify oocyst infectivity. Identifying the mechanisms involved in the virulence of *C. parvum* or its resistance to phagocytosis could facilitate the development of new therapeutic approaches in cryptosporidiosis disease.

## Supplementary material

The supplementary material of this article is available at https://www.parasite-journal.org/10.1051/parasite/2023033/olm. Video of interactions between *C. parvum* oocysts and *Acanthamoeba castellanii*. A favorable tropism of *A. castellanii* in the direction of *C. parvum* oocysts is visible. Oocysts seemed sometimes to be incorporated into free-living amebae but were always released over time.
